# Backbone 1H, 13C, and 15N chemical shift assignments for human SERF2

**DOI:** 10.1007/s12104-024-10167-5

**Published:** 2024-03-11

**Authors:** Bikash R. Sahoo, Vivekanandan Subramanian, James C.A. Bardwell

**Affiliations:** 1 Howard Hughes Medical Institute, Chevy Chase MD-20815, USA; 2 Department of Molecular, Cellular and Developmental Biology, University of Michigan, Ann Arbor MI-48109, USA; 3 College of Pharmacy, University of Kentucky, Lexington KY-40508, USA

**Keywords:** SERF, NMR, Paramagnetic relaxation enhancement, Amyloid, α-Synuclein

## Abstract

Human small EDRK-rich factor protein SERF2 is a cellular driver of protein amyloid formation, a process that has been linked to neurodegenerative diseases including Alzheimer’s and Parkinson’s disease. SERF2 is a 59 amino acid protein, highly charged, and well conserved whose structure and physiological function is unclear. SERF family proteins including human SERF2 have shown a tendency to form fuzzy complexes with misfolded proteins such as α-Synuclein which has been linked to Parkinson’s disease. SERF family proteins have been recently identified to bind nucleic acids, but the binding mechanism(s) remain enigmatic. Here, using multidimensional solution NMR, we report the ^1^H, ^15^N, and ^13^C chemical shift assignments (~ 86% of backbone resonance assignments) for human SERF2. TALOS-N predicted secondary structure of SERF2 showed three very short helices (3–4 residues long) in the N-terminal region of the protein and a long helix in the C-terminal region spanning residues 37–46 which is consistent with the helical content indicated by circular dichroism spectroscopy. Paramagnetic relaxation enhancement NMR analysis revealed that a short C-terminal region E53-K55 is in the proximity of the N-terminus. Having the backbone assignment of SERF2 allowed us to probe its interaction with α-Synuclein and to identify the residues in SERF2 binding interfaces that likely promote α-Synuclein aggregation.

## Biological context

Accumulation of misfolded proteins is a key pathological marker of many human neurodegenerative diseases. Under pathological conditions, soluble misfolded proteins self-assemble into extended linear structures called amyloid fibers. While the aggregation of misfolded proteins is unlikely to be the sole cause of many neurodegenerative diseases, a plethora of studies have demonstrated that the deposition of amyloid fibers in the form of plaques in the human brain is a neuropathological hallmark of dementia. Several biomacromolecules including proteins, membranes, and metals have been shown to modulate amyloid formation during disease progression. Small EDRK-rich factor (SERF) proteins have been identified as a driver of protein aggregation and amyloid formation under pathological conditions in vivo (Sahoo & Bardwell 2022, van Ham et al. 2010). SERF proteins appear to be present in all sequenced eukaryotic species. SERF family proteins including human SERF1a (Falsone et al. 2012; Meyer et al. 2020), SERF2 (Stroo et al. 2023), C. *elegans* MOAG-4 (van Ham et al. 2010) and yeast SERF (Meinen et al. 2019) have been shown to promote amyloid formation of proteins that have been associated with fatal neurodegenerative diseases including Alzheimer’s, Parkinson’s, and Huntington diseases.

Human SERF2 exists in four major isoforms that are produced via alternative splicing. The smallest and longest isoforms (UniProt: P84101) are 45 and 170 amino acids long but the 59-residue long SERF2 has been considered to be the canonical SERF2 sequence. SERF2 is ubiquitously expressed in 27 different human tissues and is substantially more abundant than its homologue SERF1A/B (Fagerber et al. 2014 and Wang et al. Proteomics 2015). SERF2 has been shown in a peptide microarray analysis to bind many amyloidogenic peptide sequences (Pras et al. 2021). SERF2 function has also been shown to be linked with developmental deficits and embryonic lethality (Cleverley et al. 2021; Stroo et al. 2023). While the pathological role of SERF2 is reasonably well established, its physiological function(s) is starting to be explored, we recently showed that it binds specifically to G4 quadruplex forming sequences (Sahoo et al. 2023).

Investigation of the interactions between SERF2 and its pathological binding partners namely amyloidogenic proteins or physiological G4-quadruplex binding partners might help to explain how SERF2 triggers amyloid formation. The disordered and highly dynamic nature of SERF2’s structure however poses roadblocks to understanding its mechanism of action. Here, using solution NMR methods, we have assigned the backbone and sidechain 1H, 15N and 13C resonances of SERF2 (BMRB ID: 52,141). The predicted secondary structure from NMR chemical shifts agrees with circular dichroism (CD) spectroscopy experiments that indicate that SERF2 has a disordered structure with ~ 30% helicity at 5°C that becomes more disordered at 37°C. Paramagnetic relaxation enhancement (PRE) NMR analysis showed that SERF2 N- and C-termini are in proximity and that both are involved in interaction with α-Synuclein. The chemical shift perturbation analysis has allowed us to identify SERF2 residues that are involved, directly or indirectly, in α-Synuclein interaction. SERF2 chemical shift assignments could help us to understand its role in protein aggregation and nucleic acid interaction at atomic resolution.

## Methods and experiments

### Expression and purification of SERF2

The codon optimized human SERF2 gene (59 amino acids, UniProt: P84101-1) was synthesized by Genscript and was subcloned into the pET28a His-SUMO vector as previously described (Meinen et al. 2019). The expression plasmid was transformed into *E. coli* BL21 DE3 for protein expression. 10 ml of an overnight culture of this strain was added to 1 L of fresh PEM medium containing 50 mg/L kanamycin and the cells were grown at 37°C under constant shaking until the OD_600_ reached 1. Uniformly isotope labeled ^15^N and ^15^N-^13^C SERF2 proteins for NMR studies were produced by growing cells in M9 minimal medium supplemented with 100% ^15^N NH_4_Cl (1 g/L) and ^15^N NH_4_Cl (1 g/L), D-Glucose-^13^C_6_ (4 g/L), respectively. The bacterial culture was next transferred to 20°C with continued shaking for an hour. IPTG was then added to 0.1 mM to induce protein expression overnight at 20°C. Cells were harvested by centrifugation at 5000×g for 20 min and then resuspended in 100 mL of lysis buffer (40 mM Tris-HCl, pH 8.0, 10 mM sodium phosphate, 400 mM NaCl, 20 mM imidazole, 10% glycerol, 1 tablet of cOmplete protease inhibitor (Roche), 1.25 μg/mL Dnase I (Roche) and disrupted by sonication. The cellular lysate was centrifuged for 30 min at 4°C at 36,000×g. The supernatant was collected and passed through a 5 ml HisTrap column (Cytiva), followed by a 40 ml wash using lysis buffer. The SERF2 protein was eluted four times by application of 10 ml of lysis buffer containing 500 mM imidazole. The eluates that contained the majority of the His-SUMO-SERF2 were pooled and beta-mercaptoethanol was added to 5 mM. The His-SUMO-SERF2 protein was next mixed with 10 μl of SUMO protease ULP-1 to cleave off the His-SUMO tag. The digestion mixture was dialyzed against 1 L of dialysis buffer (40 mM Tris-HCl, pH8.0, 300 mM NaCl) using a 3 kD cutoff dialysis membrane (Amicon^®^, UFC500324) at 4°C overnight. After dialysis, the digestion mixture was diluted with an equal volume of 50 mM sodium phosphate buffer pH 6.0 to adjust the NaCl concentration to 150 mM. This solution was passed through a 5 mL HisTrap column (Cytiva, 17,524,802) to remove the His-SUMO tag, resulting in the purification of an unfused SERF2 protein that contained just a single additional serine at the N-terminus. The flowthrough was suspended in buffer A (50 mM sodium phosphate, 125 mM NaCl, pH 6.0) and subjected to ion exchange for further purification using an ion exchange HiTrap SP column (Cytiva, 17,115,201). The bound proteins were eluted with buffer B (50 mM sodium phosphate, 1 M NaCl, pH 6.0) running with a linear gradient set from 2 to 45% during the 16-column volume elution. Fractions containing the majority of SERF2 were pooled and concentrated using 3 kD cutoff spin column (Millipore) to a volume of 4.5 mL. This eluate was further purified by the use of a size exclusion column HiLoad 16/60 Superdex S75 (GE Healthcare, 17,106,801) using buffer 40 mM HEPES-NaOH, pH 7.5, 150 mM NaCl. Fractions of pure SERF2 were pooled, concentrated, snap frozen in liquid nitrogen and stored at −80°C. For biophysical and NMR studies, SERF2 proteins were buffer exchanged in 20 mM d3-sodium acetate, 20 mM KCl, 2 mM EDTA pH 5.5 or 20 mM sodium phosphate, 100 mM KCl, pH 7.4 or 50 mM Tris-HCl, pH 7.4 using a 3-kDa Amicon Ultra-4 centrifugal filter (EMD Millipore, UFC800324). SERF2 concentration was determined by Pierce^™^ BCA assay kit. For a protein standard we used an A51W mutant of SERF2 whose concentration had been determined by tryptophan absorption using UV-3000 (Shimadzu) spectrometer. This mutant was expressed and purified using the same protocol as we used for the wild-type SERF2 protein.

### NMR spectroscopy

Proton NMR of 100 μM of human SERF2 protein that had been dissolved in two different buffers (20 mM d3-sodium acetate, 20 mM KCl, 2 mM EDTA pH 5.5 or 20 mM sodium phosphate, 100 mM KCl, pH 7.4) that varied in pH were collected to check the spectral quality prior to conducting 2D and 3D NMR measurements. A better resolution spectrum was obtained at pH 5.5 so this pH was used for multidimensional NMR experiments. 1.2 mM SERF2 protein was dissolved in 20 mM d3-sodium acetate buffer, 20 mM KCl, 2 mM EDTA, pH 5.5 that contained 92.5% H_2_O and 7.5% D_2_O. 280 μL of protein samples were loaded into a Shigemi NMR tube to collect 2D ^1^H, ^15^N HQSC, ^1^H, ^13^C HSQC, 3D HNCA, HNCO, HNCACB, CBCA(CO)NH, HN(CA)CO, ^15^N TOCSY-HSQC and ^15^N NOESY-HSQC spectra for backbone and sidechain assignments. NMR data were collected on an 800 MHz Bruker NEO spectrometer equipped with a 5 mm triple resonance inverse detection TCI cryoprobe at 277.1 K.

All NMR data were processed using Bruker’s Topspin 4.1.4. NMR data analysis and chemical shift assignments were done using NMRFAM-Sparky 1.47 (Lee et al. 2015). Chemical shift perturbation and peak height analysis of ^1^H, ^15^N HQSC spectrum are done using perturbation analysis programs in NMRFAM-Sparky.

PRE NMR experiments were carried out on a Bruker 800 MHz spectrometer using 280 μL of samples loaded into a Shigemi NMR tube. 60 μM of a uniformly ^15^N labelled SERF2 T2C variant that had also been labelled with S-(1-oxyl-2,2,5,5-tetramethyl-2,5-dihydro-^1^H-pyrrol-3-yl) methyl methanesulfonothioate (MTSL) was dissolved in 50 mM Tris-HCl (pH 7.4), 20 mM NaCl, in 90% H_2_O and 10% D_2_O. The MTSL spin labeling protocol used was as previously described (Sahoo et al. 2023a). 2D ^1^H, ^15^N HSQC spectra were recorded at 277.1 K in paramagnetic conditions using 16 scans, 1s delay and 128 t1 increments. The diamagnetic spectrum of the SERF2 T2C-MTSL sample was recorded after reducing the protein with 6 mM sodium ascorbate for ~ 3 h. ^1^H, ^15^N HSQC spectrum of 60 μM SERF2 mixed without and with equimolar α-Synuclein was recorded at 277.1 K with 1s delay, 16 scans and 128 t1 increments. For PRE NMR measurements, 60 μM SERF2 mixed with 60 μM α-Synuclein was used for ^1^H, ^15^N HSQC measurements in paramagnetic and diamagnetic (reduced with 6 mM sodium ascorbate prior to recording the diamagnetic spectrum) conditions.

### Extent of assignments and data deposition

**Fig. 1:**
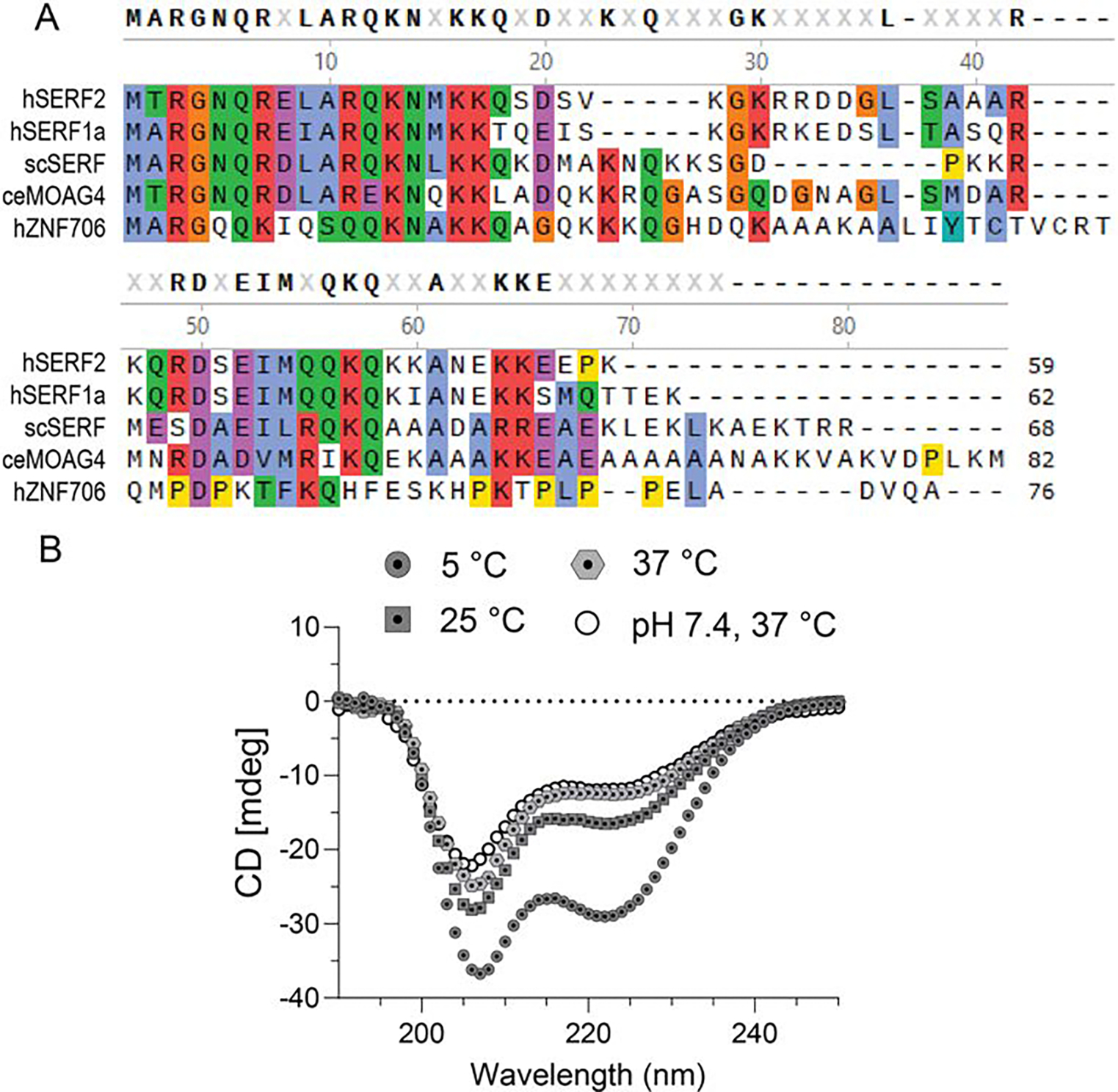
Human SERF2 is highly charged and partially disordered. (**A**) Multiple protein sequence alignment showing amino acid conservation between human (h) SERF2 and its homologs that include human SERF1a, ZNF706, yeast (sc) SERF, and *C. elegans* (ce) MOAG4. (**B**) CD spectra of 50 μM human SERF2 dissolved in 20 mM d3-sodium acetate, 20 mM KCl, 2 mM EDTA, pH 5.5 buffer at different temperatures as indicated, compared with CD spectrum of the protein in 50 mM Tris-HCl, pH 7.4 at 37°C

A multiple sequence alignment of human SERF2 with some of its homologs is shown in Fig. 1A. Human SERF2 showed 69.5% identity to human SERF1a, 51.9% identity to yeast SERF and 52.5% identity to *C. elegans* MOAG4 and 25.9% identity to the more distantly related human ZNF706. At a physiological pH of 7.4 and temperature of 37°C, human SERF2 is highly disordered as indicated by its CD spectra as it shows a prominent minimum at ~ 205 nm. As we lower the temperature, the helical content in SERF2 increases as evidenced from an increase in the CD peak at ~ 222 nm (Fig. 1B). NMR spectra collected at 37°C and pH 7.4 showed a narrow spectral distribution for SERF2 and the majority of the amide cross peaks are undetectable presumably due to line broadening (Fig. S1). When incubated under acidic conditions (pH 5.5) at 37°C, many more peaks appeared (Fig. S1). CD analysis indicates that similar overall secondary structure exists at both pH 7.4 and 5.5 at 37°C (Fig. 1B). We thus reasoned the differences in HSQC spectra at these two pH values might arise from intermediate amide proton exchange as is commonly observed for disordered proteins at high temperature (Yamaguchi et al. 2011).

**Fig. 2:**
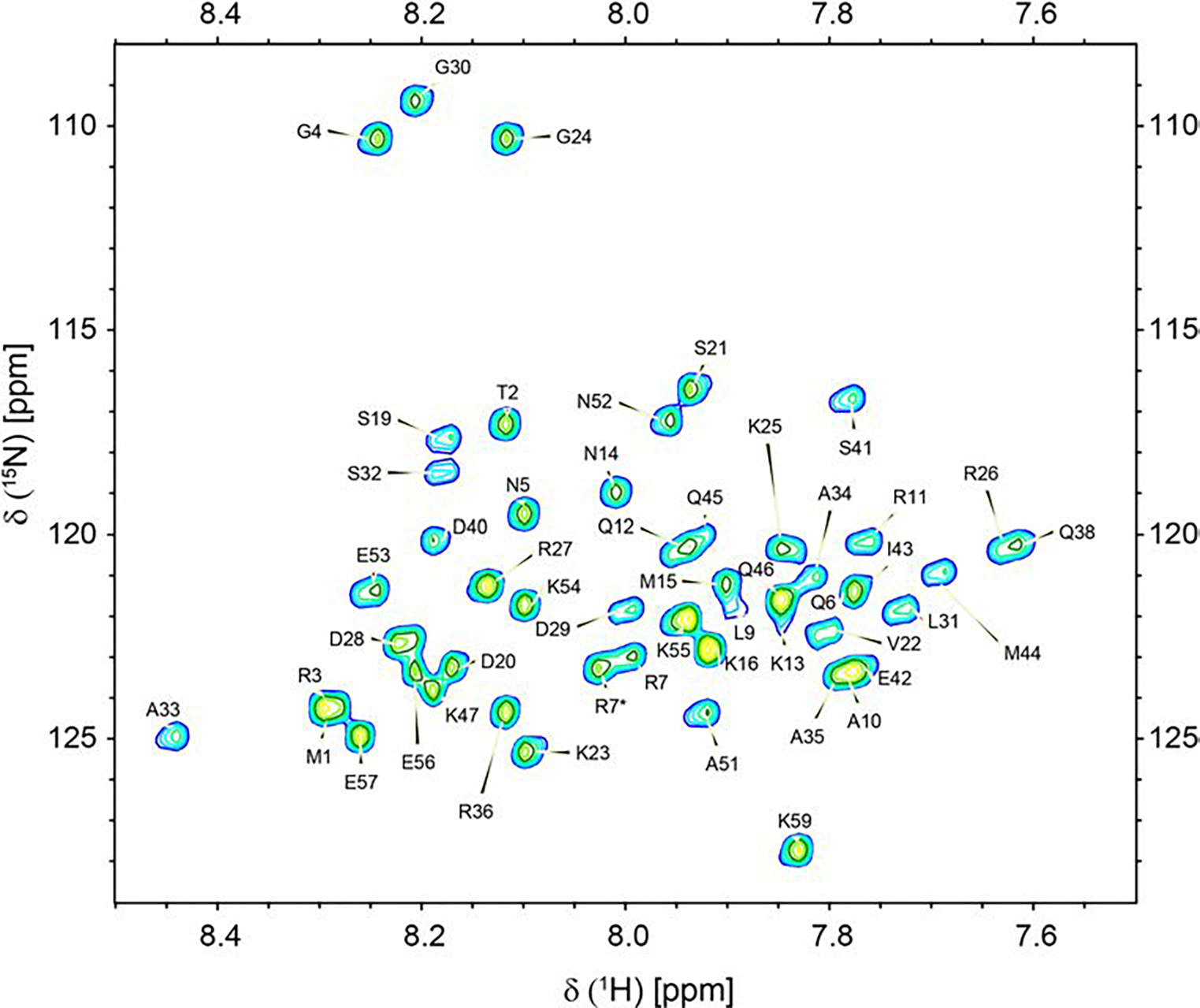
2D ^1^H, ^15^N HSQC spectrum recorded at 800 MHz 1H frequency showing the backbone N, H assignments of human SERF2 dissolved in 20 mM d3-sodium acetate, 20 mM KCl, 2 mM EDTA, pH 5.5 buffer containing 92.5% H_2_O and 7.5% D_2_O. NMR spectrum was collected on an 800 MHz Bruker spectrometer at 277.1 K

SERF2 chemical shift assignments were performed using uniformly labelled ^13^C, ^15^N human SERF2 protein that had been dissolved in acidic (pH 5.5) buffer conditions at 4°C where the majority of the peaks are well resolved (Fig. 2). The backbone assignment and sequence connectivity were achieved using 3D HNCO, HNCA, CBCA(CO)NH, HNCACB and HN(CA)CO spectra. These sequential assignments were supported by 3D ^15^N TOCSY-HSQC and ^15^N NOESY-HSQC experiments through utilizing NOE patterns and their associated ^15^N and ^1^H chemical shifts. Overlapped signals in the 2D HSQC spectrum were assigned by referring to the 3D ^15^N TOCSY-HSQC, ^15^N NOESY-HSQC, and HNCO spectra. ^1^H, ^15^N HSQC correlation peaks for 50 out of 58 non-proline residues were assigned. The unassigned SERF2 amino acid residues in the ^1^H, ^15^N HSQC spectrum are Glu8, Lys17, Gln18, Gln37, Arg39, Gln48, Lys49, and Lys50. The Pro58 residue was assigned using 3D ^15^N TOCSY-HSQC and ^15^N NOESY-HSQC experiments that showed ^H^N(Glu57)-H^δ^(Pro58) and ^α^H(Glu57)-H^δ^(Pro58) NOEs and a ^13^C^β^ chemical shift of 28.948 ppm suggestive of a trans proline conformation, which occur in the range of 31.7 ± 4 ppm. Side-chain assignments were done using 3D ^15^N TOCSY-HSQC and ^15^N NOESY-HSQC spectrum following standard procedures. The ^1^H, ^15^N, ^13^C chemical shift assignments for human SERF2 have been deposited in the BioMagResBank (http://www.bmrb.wisc.edu) under accession number 52,141. SERF2 assignment percentages include ^1^H^N^ 86% (50 out of 58 non-proline residues), ~ 84% ^15^N (50 out of 59), ^13^Cα 88% (52 out of 59), ^13^Cβ 87% (49 out of 56 non-glycine residues), and ^13^CO 83% (49 out of 59).

**Fig. 3:**
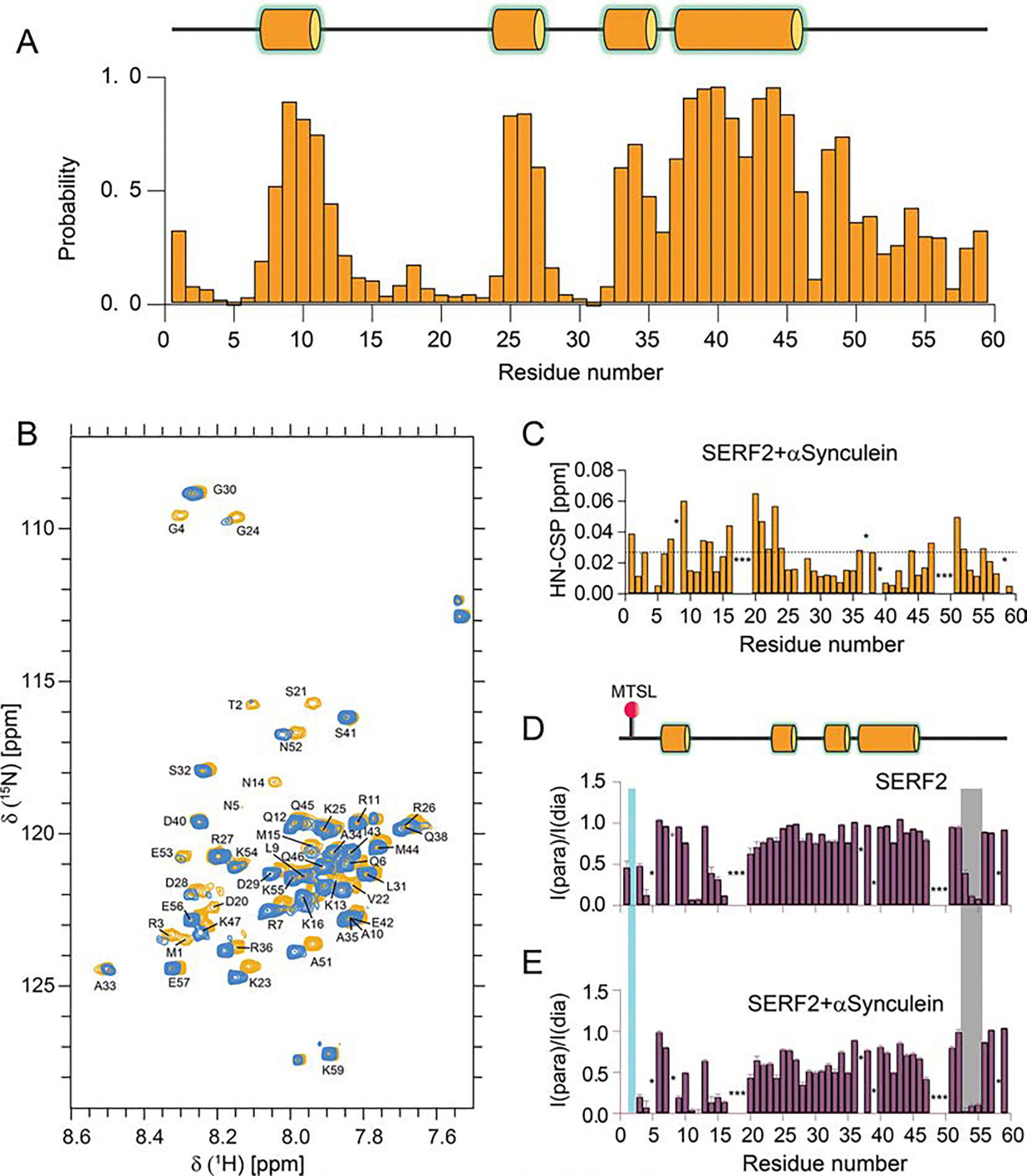
NMR binding interaction studies between SERF2 and α-Synuclein. (**A**) Secondary structure of human SERF2 predicted from backbone chemical shifts using TALOS-N, α-helices are shown as orange cylinders. (**B**) 2D ^1^H,^15^N-HSQC spectrum of 60 μM uniformly 15 N labelled SERF2 mixed without (blue) and with 60 μM of unlabeled α-Synuclein (orange) dissolved in 50 mM Tris-HCl (pH 7.4), 20 mM NaCl, 90% H_2_O and 10% D_2_O. (**C**) Chemical shift perturbation (CSP) plot (ΔHN) derived from ^1^H,^15^N-HSQC NMR spectrum shown in Fig. 2B. The average CSP is shown as a dashed line. Intensity ratio plot of 60 μM uniformly ^15^N labelled SERF2 (T2C-MTSL) in paramagnetic and diamagnetic conditions without (**D**) or mixed with an equimolar of unlabeled α-Synuclein (**E**) as indicated. The PRE effect on C-terminal residues is highlighted in grey and N-terminal PRE by T2C MTSL labeling is highlighted in cyan. ‘*’ indicates unassigned resonances. NMR spectrum was collected on an 800 MHz Bruker spectrometer at 277.1 K

We determined the amino acid specific secondary structure properties of human SERF2 from the assigned backbone chemical shifts using TALOS-N (Shen and Bax 2015). The SERF2 secondary structure as predicted by TALOS-N (Fig. 3A) correlates to the ~ 30% helical structure predicted by the BestSel program using our CD results at pH 5.5 (Micsonai et al. 2018). The N-terminal residues are highly disordered with the exception of three very short helices spanning residues R8-R11, K25-R27 and A33-A35. The short helix A33-A35 is followed by a single long helix spanning residues K37-Q46. This long helix is a conserved secondary structure motif that has been observed for the other SERF family members that include *C. elegans* MOAG4 and yeast SERF (Yoshimura et al. 2017; Meinen et al. 2019; Liu et al. 2022). The C-terminal residues K47-K59 in SERF2 are mainly disordered and predicted to be random coils in TALOS-N (Fig. 3A).

The biological role of SERF2 under pathological conditions is reported to promote amyloid aggregation both in vitro and in vivo in proteins such as α-Synuclein, a protein whose aggregation has been linked to Parkinson’s disease (Stefanis 2012). To get some structural insights into this function, we next probed the interaction between human SERF2 and α-Synuclein. These molecular interactions were monitored both at the physiological pH of 7.4, but at non-physiological temperature of 4°C since this is a temperature where major of the SERF2 ^1^H, ^15^N HSQC peaks are well dispersed and therefore detectable. α-Synuclein binding to SERF2 at equimolar concentrations induced substantial H-N chemical shift perturbations (CSPs) for several of the SERF2 residues (M1, R7, L9, Q12, K13, K16, D20-G24, R36, M44, Q45, K47, A51, N52, and K55) that all lie above the average CSP value (0.023 ± 0.01) (Fig. 3C). The binding interface between human SERF2 and α-Synuclein as indicated from the CSPs indicates a binding interface that in SERF2 spans the disordered domains in both the N- and C-termini. We note here that these observed CSPs could also be due to α-Synuclein binding mediated conformational alteration within SERF2. Two out of the three glycine residues (G4 and G24) in SERF2 in the absence of α-Synuclein showed broadened signals which are likely due to intermediate chemical exchange that occurs in these buffer conditions at pH 7.4. However, upon addition of α-Synuclein these glycine amine signals become sharp and are well resolved (Figs. 3C and S2). Both of these glycine residues are located in the N-terminal binding interface of SERF2, indicating that α-Synuclein interaction may directly modulate SERF2 structure and dynamics, but this hypothesis awaits further experimental confirmation.

Mutational studies on SERF family proteins have been interpreted to indicate that N-terminal residues in SERF form the key binding interface with amyloid proteins, a result consistent with our NMR data. However, our NMR experiments indicate that several C-terminal residues are also involved in interaction with α-Synuclein. To better understand the role of these C-terminal residues in α-Synuclein interaction, we performed PRE NMR. PRE NMR analysis of SERF2 without α-Synuclein in paramagnetic and diamagnetic conditions showed a strong PRE effect of a N-terminally located MTSL probe (T2C MTSL) on three C-terminal residues E53, K54 and K55, and also several N-terminal residues in the region 11–16 (Fig. 3D). This result suggests the disordered N-terminus of SERF2 (residues ~ 1–16) is likely located in close proximity to SERF2’s C-terminal region (residues 53–55). Interestingly, SERF2, when mixed with α-Synuclein showed a greater degree of PRE effect in regions spanning residues 20–35, a region that shows only a minor PRE effect in the absence of α-Synuclein (Fig. 3E). The PRE effects observed for the C-terminal SERF2 residues E53, K54 and K55 were enhanced upon α-Synuclein binding suggesting that these C-terminus residues are directly or indirectly engaged in α-Synuclein binding. In summary, our NMR analysis suggests that the N-terminal domain in SERF2 triggers α-Synuclein binding which in turn affect the SERF2’s overall conformation.

## Supplementary Material

supplement #1

Supplement #2

## Data Availability

The 1H, 13C, and 15N chemical shifts of human SERF2 has been deposited to the BioMagResBank (www.bmrb.wisc.edu) under the accession number BMRB-52141.
