# Supplementary material for: Backbone 1H, 13C, and 15N chemical shift assignments for human SERF2
Source: Biomol NMR Assign. Author manuscript; Available in PMC 2025 May 23. (PMC12022958; doi:10.1007/s12104-024-10167-5)
Supplement: supplement #1 [file NIHMS2067100-supplement-supplement__1.docx]

**Supporting Information**

**Backbone 1H, 13C, and 15N chemical shift assignments for human SERF2**

Bikash R. Sahoo,^1,2*^ • Vivekanandan Subramanian^3^ • James C.A. Bardwell^1,2*^

^1^Howard Hughes Medical Institute

^2^Department of Molecular, Cellular and Developmental Biology, University of Michigan, Ann Arbor, MI-48109, USA

^3^College of Pharmacy, University of Kentucky, Lexington, KY-40508, USA

*To whom correspondence should be addressed

**Abstract**

Human small EDRK-rich factor protein SERF2 is a cellular driver of protein amyloid formation which has been linked to many different neurodegenerative diseases including Alzheimer’s and Parkinson’s disease. SERF2 though tiny (59 residues) and highly charged, its structure and physiological function remain unexplored. SERF family proteins including human SERF2 is shown a tendency to form fuzzy complexes with misfolded proteins such as α-Synuclein which has been linked to Parkinson’s disease. SERF family proteins have been recently identified to bind nucleic acids, but the binding mechanism(s) remain enigmatic. Here, using multidimensional solution NMR, we report the ^1^H, ^15^N, and ^13^C chemical shift assignments (~86 % of backbone resonance assignments) for human SERF2. TALSO-N predicted secondary structure of SERF2 showed three short-helix (3-4 residues long) at N-terminus and a long (region 37-46) which correlates to SERF2’s helical structure observed by circular dichroism spectroscopy. Paramagnetic relaxation enhancement (PRE) NMR analysis revealed a short C-terminal region E53-K55 is spatially oriented in the proximity of the N-terminus. The backbone assignment of SERF2 led us to probe its interaction with α-Synuclein and identifying key binding interfaces in SERF2 that promote α-Synuclein aggregation.

Table of Contents

Supplementary Figure S1: Human SERF2 proton NMR spectral quality assessment at different temperatures and pH values for multidimensional measurements.

Supplementary Figure S2: Peak height ratios of SERF2 without or with the addition of an equimolar α-Synuclein, ratios obtained from the 1H, 15N HSQC experiment shown in Figure 2B.


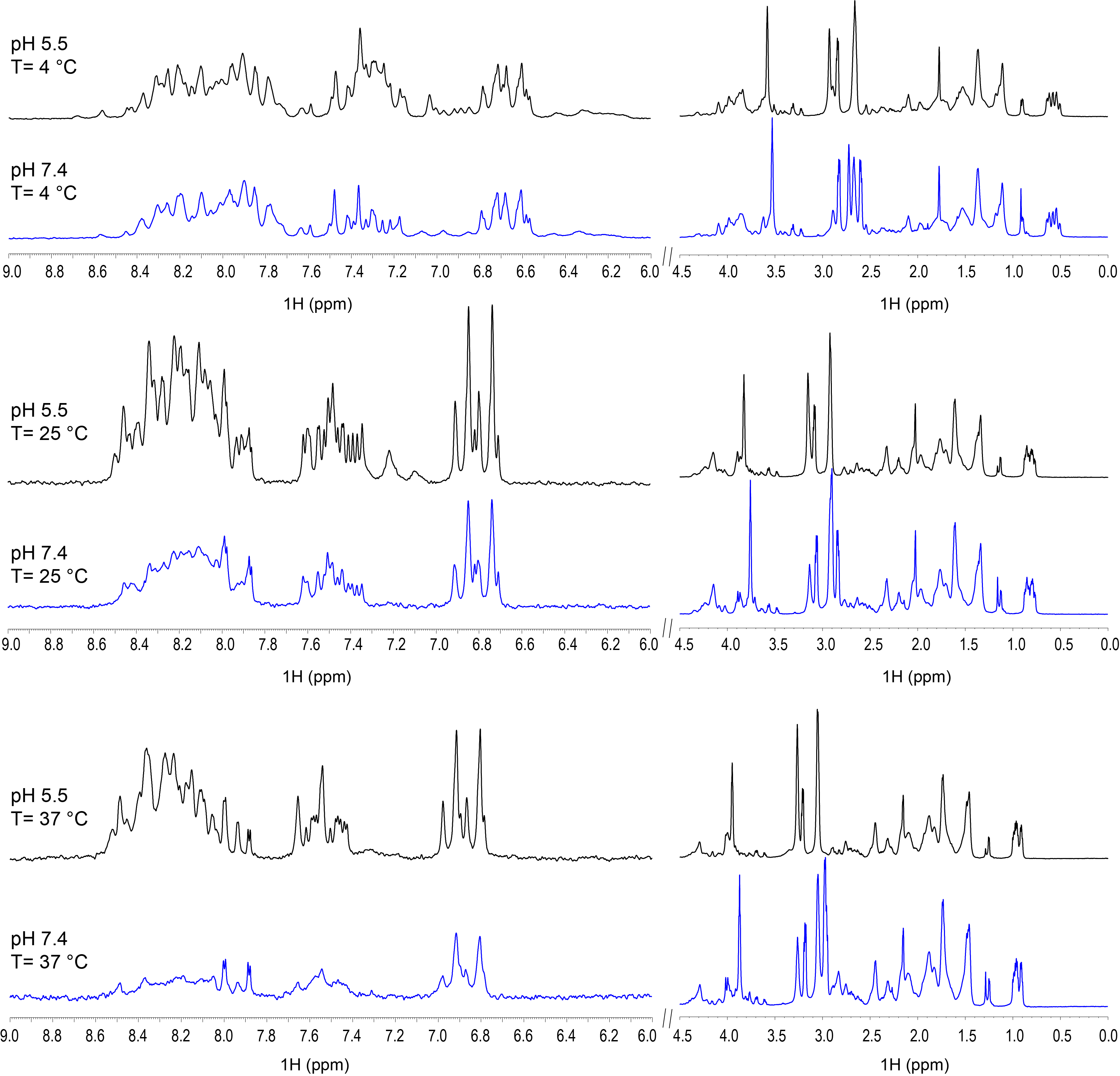


Figure S1**:** 1D proton NMR spectra of 100 µM human SERF2 at the different indicated temperatures dissolved in either 20 mM d3-sodium acetate, 100 mM KCl, pH 5.5 (black), or 20 mM sodium phosphate, 100 mM KCl, pH 7.4 (blue). NMR spectra were recorded on a Bruker 800 MHz instrument.


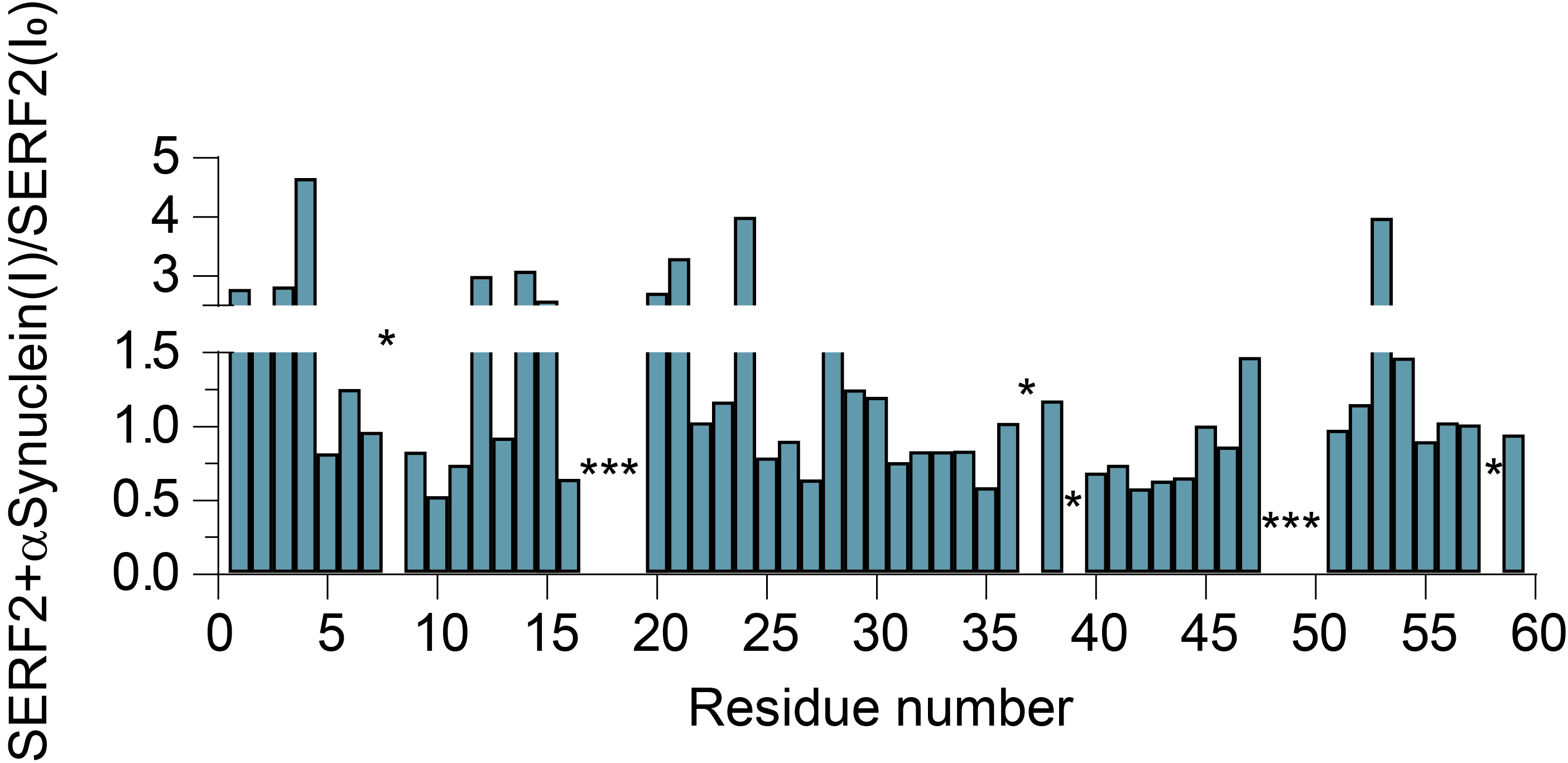


Figure S2**:** 1H, 15N HQSC peak height ratio of uniformly 15N labelled 60 µM SERF2 mixed with an equimolar amount of unlabeled α-Synuclein (I) to the values seen for 60 µM 15N labelled SERF2 in the absence of α-Synuclein (I_0_). ‘*’ indicates unassigned resonances. The corresponding 1H, 15N HQSC spectra are shown in Figure 2B.

1D proton NMR spectra of 100 µM human SERF2 at different temperatures as indicated dissolved in 20 mM d3-sodium acetate, 100 mM KCl, pH 5.5 (black), or 20 mM sodium phosphate, 100 mM KCl, pH 7.4 (blue). NMR spectra were recorded on a Bruker 800 MHz instrument.
